# Development and application of an indirect ELISA for detection of antibodies against emerging atypical porcine pestivirus

**DOI:** 10.1186/s12985-024-02330-0

**Published:** 2024-03-04

**Authors:** Hao Song, Xiaowei Gao, Jing Li, Xinying Dong, Yanhui Fu, Lina Shao, Jiaoer Zhang, Hua-Ji Qiu, Yuzi Luo

**Affiliations:** State Key Laboratory for Animal Disease Control and Prevention, Harbin Veterinary Research Institute, Chinese Academy of Agricultural Sciences, 678 Haping Road, Harbin, 150069 China

**Keywords:** Atypical porcine pestivirus, E2 protein, Indirect ELISA, Seroprevalence

## Abstract

**Background:**

Atypical porcine pestivirus (APPV) is a newly discovered swine pestivirus, which can cause congenital tremor and high mortality in newborn piglets and subclinical infection in adult pigs, leading to significant impacts on the pig industry. Currently, there is no approved serological method to assess APPV infection status in pig farms.

**Methods:**

In this study, the envelope glycoprotein E2 of APPV was highly expressed in suspension HEK293 cells, and further an indirect enzyme-linked immunosorbent assay based on the recombinant E2 protein (E2-iELISA) was developed and evaluated.

**Results:**

The reaction parameters of the E2-iELISA were optimized, and the cutoff value was determined to be 0.2 by analyzing S/P values of 165 negative sera against APPV that were confirmed by virus neutralization test (VNT). Specificity test showed that the method had no cross-reaction with other common swine viruses. The E2-iELISA was evaluated using a panel of swine sera, and showed high sensitivity (113/120, 94.2%) and specificity (65/70, 92.9%), and the agreement rate with VNT was 93.7% (178/190). Subsequently, the E2-iELISA was utilized to investigate the seroprevalence of APPV in pig herds of China. When detecting 1368 pig serum samples collected from nine provinces in China, the overall seroprevalence of APPV was 73.9% (1011/1368).

**Conclusion:**

Our findings suggest that the E2-iELISA is specific and sensitive, and could be a valuable tool for serological surveillance of APPV infection in pigs.

## Introduction

Pestiviruses belong to the family *Flaviviridae* and comprise several economically important animal pathogens that cause severe economic losses to the animal husbandry, such as classical swine fever virus (CSFV) and bovine viral diarrhea virus (BVDV), etc. The species of pestiviruses have gradually increased over the past years, and currently the genus *Pestivirus* includes at least eleven species (*Pestivirus A* to *K*) [1]. Atypical porcine pestivirus (APPV), which was discovered by metagenomic sequencing of serum samples from pigs infected with porcine reproductive and respiratory syndrome virus (PRRSV) in USA in 2015 [2], is classified as *Pestivirus K* [1]. Subsequently, APPV was found to be associated with congenital tremor in newborn piglets by two independent studies via the inoculation of pregnant sows with infectious animal materials [3, 4]. Similar to other pestiviruses including CSFV, BVDV, and border disease virus (BDV), APPV has an RNA genome of approximately 11–12 kb in size, containing a single large open reading frame (ORF). The ORF encodes a polyprotein composed of approximately 3635 amino acids that are processed into four structural proteins (C, E^rns^, E1, and E2) and eight non-structural proteins (N^pro^, p7, NS2, NS3, NS4A, NS4B, NS5A, and NS5B) [2]. The polyprotein sequence of APPV has about 68% homology with that of bat pestivirus, and less than 40% identity with that of CSFV, BVDV, or BDV [2]. In addition, the current data provide no evidence that the presence of the APPV genome or APPV-specific antibodies interfere with the diagnostic tests routinely used for CSF [5].

APPV is widely distributed around the world and has been detected in domestic pigs in Americas [2, 6, 7], Europe [8–10], and Asia [11–19]. The mortality has been reported to be as high as 30% among APPV-infected piglets [9], seriously affecting the pig industry. APPV was first reported in Guangdong Province of China in 2016 [13], and subsequently it has been detected in southern and northern China, including Sichuan, Guangxi, Anhui, Hubei, Henan, and Heilongjiang, etc. [15–19]. A serological survey of APPV among apparently healthy domestic pigs in different parts of Europe and Asia conducted by a German research group, revealed that 60% (880/1460) of the pigs displayed intermediate to high antibody levels against APPV [8]. In addition, a high APPV seroprevalence has been demonstrated in wild boars in Germany (52%, 237/456) [20] and Sweden (72%, 433/595) [21]. By far, few serological investigations on APPV have been reported among domestic pigs and wild boars in China.

The wide geographic distribution of APPV-infected pigs, coupled with frequent international trade and travel, has substantially increased the risk of introduction and transmission of APPV to nonepidemic areas. Thus, there is an urgent need to develop diagnostic assays that are economically viable, robust, sensitive, and specific for detecting potential APPV infections in susceptible hosts, especially in nonepidemic areas. So far, several polymerase chain reaction (PCR) assays have been reported for detecting the APPV RNA, including reverse transcription quantitative real-time PCR (RT-qPCR), droplet digital PCR (ddPCR), and multiplex RT-qPCR [22–24]. Given that antibodies appear early in the viral infection and can last for months or even years, and currently there is no commercial vaccine available for APPV, serological testing is a better way to assess the infection status of animals (infected or non-infected) than nucleic acid testing. For pestiviruses, virus neutralization test (VNT) is the gold standard for detection of antiviral antibodies. However, VNT requires a biocontainment facility that can handle live viruses and is time-consuming, complex to operate, and inappropriate for large-scale screening.

Enzyme-linked immunosorbent assay (ELISA) is widely used throughout the world because of its simplicity, high throughput, safety, and rapidity. So far, there is no commercially available serological diagnostic kits for APPV. Several groups have reported ELISAs for detecting antibodies against APPV. Postel et al. (2017) developed an indirect ELISA (iELISA) based on the APPV E^rns^ protein expressed in *Leishmania tarentolae* [5], however, the diagnostic performance, including sensitivity and specificity, has not been rigorously evaluated, although which has been applied in some studies [8, 20, 25, 26]. Arruda et al. (2022) evaluated the potential of the established iELISA based on the APPV E^rns^, E2, or NS3 proteins using the samples of known infection status [27]. A Chinese research group established an iELISA based on the recombinant E2 or E^rns^ protein of APPV expressed in the form of inclusion bodies in *E. coli*. Regrettably, they used only 20 sera to determine the cutoff value and 50 sera from one region to evaluate the ELISA, and all the tested sera were not confirmed by any other assay [28, 29]. In short, the above studies have not systematically evaluated the established ELISA.

Since the eukaryotic expression system has the advantages of protein processing, folding, and post-translational modification that the prokaryotic system does not have, it is more conducive to the expression of highly glycosylated envelope proteins to ensure their biological activities. In this study, the APPV E2 protein was efficiently expressed in the eukaryotic expression system (HEK293 cells) with the serum-free suspension culture technology. Subsequently, an indirect ELISA based on the recombinant E2 protein (E2-iELISA) was established and evaluated. This method was further used to investigate the seroprevalence of APPV in pigs in China.

## Materials and methods

### Plasmids, cells, viruses, and serum samples

The lentiviral vector pLVX-IRES-ZsGreen1 and the helper plasmids psPAX2 and pMD2.G were purchased from Addgene (USA). Both human embryonic kidney (HEK) 293T cells and porcine kidney (PK-15) cells were cultured in Dulbecco’s modified Eagle’s medium (DMEM) (Gibco, Beijing, China) supplemented with 10% fetal bovine serum (FBS) (Sigma-Aldrich, St. Louis, MI, USA), 100 μg/mL streptomycin, and 100 IU/mL penicillin at 37 °C in 5% CO_2_. Suspension HEK293 cells were cultured in CD 293 serum-free Medium (BasaIMedia, Shanghai, China). The APPV China/HLJ491/2017 strain (GenBank accession no. OQ032517) was propagated in PK-15 cells as described previously [19].

The sera from a pig experimentally infected with APPV China/HLJ491/2017 strain (unpublished data) and from a specific pathogen free (SPF) pig were used as positive and negative control, respectively. Both sera were confirmed by VNT and immunofluorescence assay (IFA). The anti-BVDV swine sera (4) used in this study were provided by the EU Reference Laboratory for CSF, Hannover, Germany; antisera against PRRSV (4), CSFV (4), pseudorabies virus (PRV) (5), and porcine circovirus type 2 (PCV2) (3) were collected from corresponding immunized pigs and stored in Harbin Veterinary Research Institute, Chinese Academy of Agricultural Sciences.

A total of 1368 clinical serum samples were collected for passive surveillance by pig farmers or local veterinarians during 2017 to 2021 from 53 pig herds in different provinces of China, including Fujian (116 samples from 4 pig farms), Beijing (112 samples from 3 pig farms), Heilongjiang (172 samples from 8 pig farms), Shanxi (155 samples from 5 pig farms), Inner Mongolia (214 samples from 5 pig farms), Hebei (185 samples from 9 pig farms), Sichuan (178 samples from 6 pig farms), Hubei (44 samples from 2 pig farms) and Liaoning (192 samples from 11 pig farms).

### Construction of the recombinant plasmid harboring the APPV *E2* gene

The E2-coding region of the APPV China/HLJ491/2017 strain (GenBank accession no. OP617199) was optimized based on the synonymous codon bias of mammals. Thereafter, the codon-optimized E2 gene with *Eco*RI and *Xho*I restriction sites, N-terminal signal peptide [30], and a C-terminal 6 × His tag was synthesized by Beijing Liuhe BGI Co., Ltd. (Beijing, China). Subsequently, the gene was subcloned into the lentiviral expression vector pLVX-IRES-ZsGreen1 to generate the expression plasmid pLVX-APPV-E2. All plasmids were verified by sequencing.

### Establishment of a cell line stably expressing the APPV E2 protein

HEK293T cells were plated into a 10-cm cell culture dish to 80 to 90% confluence. One day later, the cells were co-transfected with 15 μg of the expression plasmid pLVX-APPV-E2, along with 5 μg and 10 μg of the helper plasmids pMD2.G, and psPAX2, respectively. At 48 h post-transfection, the supernatant of the cell culture was harvested and concentrated, and subsequently the suspension HEK293 cells were transduced with the recombinant lentiviruses, resulting in a recombinant suspension cell line HEK293-APPV-E2. The expression of the recombinant E2 protein in the transduced cells was examined by Western blotting analysis.

### Western blotting analysis

Western blotting analysis was performed as described previously [31]. Briefly, the supernatant of cell culture collected at 96 h post-transduction (hpt) was incubated with Ni2^+^ resin overnight, and then was subjected to 12.5% sodium dodecyl sulfate–polyacrylamide gel electrophoresis (SDS-PAGE). After electrophoresis, the separated protein band was electro-transferred onto a nitrocellulose membrane using a semidry blotter (Bio-Rad). The membrane was blocked with 5% skim milk in PBS overnight at 4 °C and further probed with anti-His-tag monoclonal antibody (MAb) (1:1000). The membrane was then washed with PBS containing 0.1% Tween 20 (PBST) and incubated with IRDye 800CW secondary antibodies (1:10,000) for 45 min at room temperature. Finally, the protein bands were visualized using an Odyssey infrared imaging system (LI-COR Biosciences).

### Expression and purification of the recombinant E2 protein

The HEK293-APPV-E2 cell line was cultured for eight days and the supernatant of cells was harvested and filtered through a 0.45-μm pore-sized membrane. The recombinant E2 protein was purified through Ni2^+^ affinity chromatography according to the manufacturer’s guidelines (GE Healthcare, USA). The purified protein was separated with 12.5% SDS-PAGE and observed after Coomassie brilliant blue staining. The protein concentration was determined using a BCA assay kit according to the manufacturer’s manual (Tiangen, Beijing, China).

### VNT and IFA

Positive or negative sera against APPV were determined by VNT as previously described [26] with some modification. Briefly, the swine serum samples were inactivated for 30 min at 56 °C and tested in a two-fold dilution series (starting from 1:5). The diluted samples were mixed with the equal volume of 200 TCID_50_ of APPV and incubated at 37 °C for 1 h. The antibody-virus mixtures were then added to the 96-well plates seeding PK-15 cells and incubated for 72 h at 37 °C. Thereafter, the 96-well plates were fixed with pre-chilled absolute ethanol for 30 min at − 20 °C, and the anti-APPV-E2 MAb (diluted 1:300 in 5% bovine serum albumin [BSA]) was incubated with the cells at 37 °C for 2 h. The cells were washed and incubated at 37 °C for 1 h with Alexa Fluor 488-conjugated anti-mouse IgG (Invitrogen, USA) (diluted 1:300 in 5% BSA). Finally, after washing with PBST, 50% glycerol was added into each well. The results were recorded under the fluorescence microscope (Nikon TE200, Japan). The neutralizing antibody (NAb) titers of the sera against APPV were expressed as the reciprocal of the highest serum dilution that inhibited virus infection in 50% of the wells [32].

### Development and optimization of the E2-iELISA

Ninety-six-well ELISA plates were coated with 100 μL of the purified recombinant E2 protein diluted in carbonate-bicarbonate buffer (pH = 9.6) and incubated at 4 °C over-night. After washing with PBST, the plates were blocked at 37 °C for 2 h with 180 μL of Blocking buffer (Surmodics, USA). Subsequently, serum samples were diluted in StabilZyme SELECT Stabilizer (Surmodics, USA) (1:100) and incubated at 37 °C for 1 h, and then the plates were washed with PBST. Thereafter, the plates were incubated with 100 μL of the horseradish peroxidase (HRP)-conjugated rabbit anti-pig IgG (Sigma-Aldrich, USA) diluted in StabilZyme SELECT Stabilizer (1:20,000) for 1 h at 37 °C. After washing with PBST, the bound antibodies were detected with TMB (100 μL/well) (Sigma-Aldrich, USA) at room temperature. The reaction was terminated by adding 50 μL of 2 M H_2_SO_4_ to each well and the optical density (OD) values at 450 nm were read using a microplate reader (Biotek, USA).

To establish an efficient ELISA, the optimal concentration of the coating antigen and the optimal serum dilution were determined by the checkerboard titration method according to the above basic conditions. Briefly, the purified E2 protein was coated to the plate in concentrations of 0.625–10 μg/mL. Positive or negative sera against APPV identified by VNT were diluted at 1:50 to 1:400. Next, HRP-conjugated rabbit anti-pig IgG (Sigma-Aldrich, USA) was diluted at 1:5000 to 1:40,000 to determine the optimal dilution. Each sample was analyzed in duplicates.

### Evaluation of the E2-iELISA

The cutoff value of the E2-iELISA was determined as described previously with some modifications [33]. 165 swine sera confirmed to be negative against APPV by VNT were tested using the E2-iELISA as described above to determine the cutoff value. S/P value: (OD_450nm_ of samples to be tested—average OD_450nm_ of negative samples)/(average OD_450nm_ of positive samples—average OD_450nm_ of negative samples), and standard deviations (SD) of the negative sera were calculated. The cutoff value = average S/P value of negative sera + 3 SD. To determine the specificity of the E2-iELISA, antisera against PRRSV, PCV2, CSFV, PRV, and BVDV were tested. For repeatability analysis, intra- and inter-assay coefficient of variation (CV) was evaluated.

To evaluate the diagnostic performance of the E2-iELISA, the assay was used to detect 190 clinical pig sera and compared with the results of VNT. Receiver operating characteristic (ROC) curve analysis was performed using MedCalc Software version 12.2.1 and relative sensitivity and specificity was determined. The area under the curve (AUC) is considered: non-informative (AUC ≤ 0.5), low accurate (0.5 < AUC ≤ 0.7), moderately accurate (0.7 < AUC ≤ 0.9), highly accurate (0.9 < AUC < 1), or perfect (AUC = 1).

### Preliminary investigation of the seroprevalence of APPV in the field by the E2-iELISA

A total of 1368 clinical pig serum samples collected during 2017–2021 from nine provinces of China (Hebei, Fujian, Sichuan, Shanxi, Beijing, Hubei, Heilongjiang, Inner Mongolia, and Liaoning) were detected by the E2-iELISA.

### Statistical analysis

The data were analyzed using GraphPad Prism version 8.3.0.

## Results

### Expression, purification, and identification of the recombinant APPV E2 protein

**Fig. 1:**
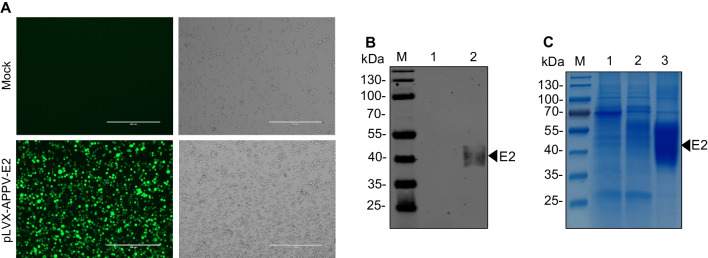
Expression of the APPV E2 protein in mammalian suspension HEK293 cells. **A** Suspension HEK293 cells were transduced with the lentiviral particles containing the recombinant plasmid pLVX-APPV-E2. The green fluorescence of cells was used as an indicator of protein expression, and mock-transduced suspension HEK293 cells were used as a negative control. The scale bar is 400 μm. **B** Western blotting analysis of the recombinant E2 protein using anti-His-tag MAb. M, protein marker; lane 1, supernatants collected from suspension HEK293 cells transduced with the empty vector at 96 h; lane 2, HEK293-APPV-E2 cell line culture supernatant at 96 h post-transduction. **C** SDS-PAGE analysis of the purified recombinant E2 protein. M, protein marker; lane 1, supernatants collected from suspension HEK293 cells transduced with the empty vector; lane 2, culture supernatants of the recombinant suspension HEK293-APPV-E2 cell line; lane 3, purified recombinant E2 protein

The lentiviral expression vector pLVX-IRES-ZsGreen1 were used to produce foreign protein, which can simultaneously express foreign protein and green fluorescent protein (GFP) in mammalian cells. Thus, the expression of the recombinant APPV E2 protein in suspension HEK293 cells could be monitored using GFP (Fig. 1A). Compared with the pLVX-IRES-ZsGreen1 empty vector, a specific protein with a molecular weight of approximately 40 kDa was detected by Western blotting using anti-His-tag MAb in the culture supernatant of HEK293-APPV-E2 cell line at 96 hpt (Fig. 1B). The recombinant E2 protein was purified by affinity chromatography of the culture supernatant of HEK293-APPV-E2 cell line collected at eight day and subjected to SDS-PAGE. The results showed that there was a diffuse band around 40 to 55 kDa, which was speculated to be the result of glycosylation modification of the recombinant E2 protein at different levels (Fig. 1C). The obtained HEK293-APPV-E2 suspension cell line was passaged for 20 consecutive generations, and the recombinant E2 protein could still be stably and efficiently expressed (data not shown), indicating that the HEK293 suspension cell line stably expressing the recombinant E2 protein was established.

### Optimization of the E2-iELISA

**Fig. 2:**
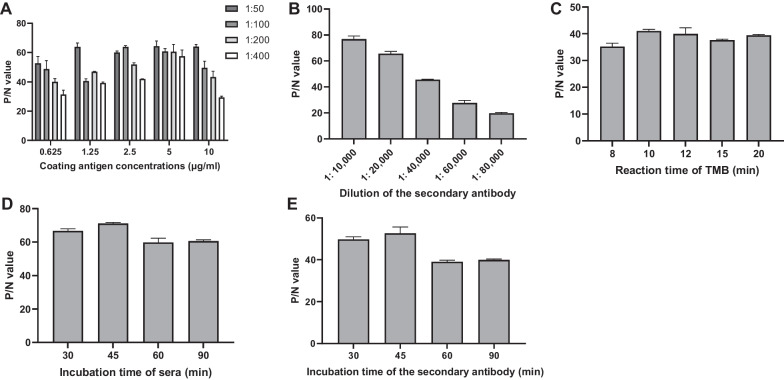
Optimization of working conditions of the E2-iELISA. **A** The checkerboard titration was performed to determine the optimal concentration of the coating antigen and serum dilution. **B** and **C** Optimization of the secondary antibody dilution and reaction time of TMB. **D** and **E** Optimization of the incubation time of sera and the secondary antibody

The E2-iELISA was optimized, and the optimal working conditions were determined as follows. As shown in Fig. 2, the concentration of coated antigen was 2.5 μg/mL, the dilutions of sera and secondary antibody were 1:100 and 1:20,000, respectively. The optimal reaction times for sera, secondary antibody, and TMB solution were 45, 45, and 10 min, respectively.

### Performance of the E2-iELISA

**Fig. 3:**
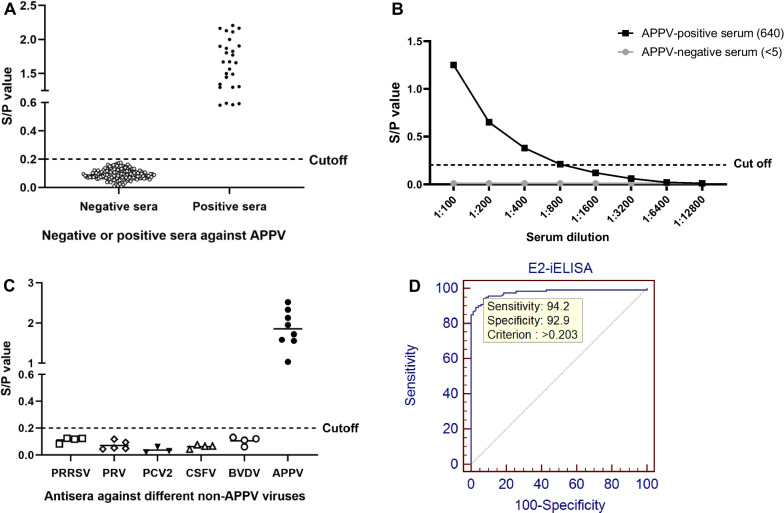
Evaluation of the E2-iELISA. **A** Determination of the cutoff value of the E2-iELISA. The cutoff value was determined by the formula (average S/P value + 3SD) using negative sera (*n* = 165) against APPV. Anti-APPV sera were served as positive controls. **B** Evaluation of the sensitivity of the E2-iELISA using serially diluted swine sera with known anti-APPV NAb titers. The numbers in brackets indicate the NAb titers. **C** Evaluation of the specificity of the E2-iELISA using a panel of swine antisera against serval non-APPV viruses, including BVDV (*n* = 4), PRV (*n* = 5), PCV2 (*n* = 3), PRRSV (*n* = 4), and CSFV (*n* = 4). **D** Evaluation of the diagnostic performance of the E2-iELISA by ROC curve analysis using MedCalc Software when detecting 70 negative and 120 positive sera against APPV verified with VNT

In total, 165 APPV-seronegative samples verified by VNT were tested by the E2-iELISA. As shown in Fig. 3A, the mean S/P value of these negative sera was 0.095, and the SD of these samples was 0.037. Therefore, the cutoff value of the E2-iELISA was calculated to be 0.2. Samples with S/P values greater than the cutoff value was considered positive.

The sensitivity of the E2-iELISA was evaluated using an anti-APPV serum with known NAb titer (640). The results showed that the E2-iELISA titer of the serum was equal to or greater than the corresponding NAb titer, which indicated a high sensitivity of the E2-iELISA (Fig. 3B). The specificity of the E2-iELISA was evaluated by testing positive sera against PRRSV, PCV2, CSFV, PRV, or BVDV. As shown in Fig. 3C, no cross-reaction between the recombination E2 protein and these sera was observed. In the repeatability test, three positive sera against APPV and one negative serum against APPV were used to evaluate the intra- and inter-assay CVs of the E2-iELISA, which were 0.20–5.57% and 4.66–7.07%, respectively (Table 1).

**Table 1:** Results of the repeatability test of the E2-iELISA

Sample number	Intra-assay variability		Inter-assay variability	
	Mean ± SD	CV (%)	Mean ± SD	CV (%)
1	3.371 ± 0.188	5.57	3.121 ± 0.145	4.66
2	2.167 ± 0.124	3.67	1.950 ± 0.114	5.85
3	0.710 ± 0.027	0.81	1.161 ± 0.082	7.07
4	0.121 ± 0.007	0.20	0.080 ± 0.004	4.86

### Agreement between the E2-iELISA and VNT

A total of 190 field swine sera identified by VNT, including 70 negative and 120 positive sera against APPV, were used to evaluate the E2-iELISA, in comparison with VNT. The ROC curve analysis showed that the E2-iELISA are highly accurate [AUC = 0.977, 95% confidence interval (CI) 0.945 to 0.993] and displayed high sensitivity (113/120, 94.2%) (95% CI 88.4 to 97.6%) and specificity (65/70, 92.9%) (95% CI 84.1 to 97.6%) (Fig. 3D), and the agreement rate with VNT was 93.7% (178/190) (Table 2).

**Table 2:** Comparison of the E2-iELISA with VNT for detection of a panel of swine sera

E2-iELISA	VNT		Total
	+	−	
+	113	5	118
−	7	65	72
Total	120	70	190
Agreement (%)	94.2	92.9	93.7

+ , positive sera against APPV; − , negative sera against APPV

### Detection of anti-APPV antibodies in field serum samples by the E2-iELISA

A total of 1368 serum samples collected from different regions in China during 2017 to 2021 were tested by the E2-iELISA for retrospective investigation of APPV infection. As shown in Tables 3 and 4, the seropositive rate of APPV in different provinces were 64.04–86.98%, except Hubei (11/44, 25%), those from 2017 to 2021 were 61.63 to 89.36%, and the overall seroprevalence was 73.9% (1011/1368).

**Table 3:** Seroprevalence of APPV in pig farms from different regions of China by the E2-iELISA

Province	Pig farms	No. of samples	No. of positive	Prevalence (%)
Fujian	4	116	92	79.31
Beijing	3	112	89	79.46
Heilongjiang	8	172	137	79.65
Shanxi	5	155	117	75.48
Inner Mongolia	5	214	161	75.23
Hebei	9	185	123	66.49
Sichuan	6	178	114	64.04
Hubei	2	44	11	25.00
Liaoning	11	192	167	86.98
Total	53	1368	1011	73.90

**Table 4:** Seroprevalence of APPV in pig farms of different years in China by the E2-iELISA

Year	Total sample	No. of positive	Prevalence (%)
2017	430	265	61.63
2018	400	294	73.50
2019	184	154	83.70
2020	166	130	78.31
2021	188	168	89.36
Total	1368	1011	73.90

## Discussion

Pestiviruses are important pathogens known to cause significant economic losses to the animal husbandry. Newly discovered swine pestiviruses include Bungowannah virus [34], Linda virus [35], and APPV [2]. Bungowannah virus and Linda virus have only been found in Australia [36] and Austria [37], where the initial disease outbreaks occurred, while APPV has been reported all over the world and confirmed to be the main cause of congenital tremor types A-II [3, 4]. Given the widespread of APPV and the serious threat to pig farms, it is critical to understand the prevalence of APPV and establish effective diagnostic methods for disease control. Currently, there are no commercially available serological test kits for APPV. Therefore, we aimed to develop an E2-iELISA, evaluate its diagnostic performance, and use this assay to investigate the prevalence of APPV in pig farms in China.

The key to developing a specific ELISA for detecting viral antibodies is to select an appropriate viral antigen. Upon pestivirus infection, antibodies are produced against the E2, E^rns^, and NS3 proteins [38–40]. It has been shown that E2 is the major antigen that induces neutralizing antibodies against APPV [26], therefore, which is suitable target for serological diagnosis of APPV. However, the E2 protein is an envelope glycoprotein with a high degree of glycosylation modification. A previous study has shown that the lack of protein glycosylation modification in prokaryotic expression system may affect the immunogenicity of the antigen [27]. Compared with the prokaryotic expression system, the mammalian cell expression system enables correct folding and post-translational processing of the expressed protein, including glycosylation, making the recombinant protein structurally and functionally similar to its natural form. Therefore, we chose HEK293 cells to express the recombinant APPV E2 protein, since the cells are easy to be transfected and capable of adapting to and growth in suspension cultures, resulting in the high-level expression of the recombinant protein. In addition, establishment of a suspension cell line expressing the recombinant E2 protein is in line with the development of commercial ELISA kits, and has the characteristics of convenience and high yield.

Subsequently, an iELISA based on the recombinant E2 protein was developed and evaluated using a panel of swine sera, with the expected results. The E2 protein coding sequence is derived from the China/HLJ491/2017 strain, which shows high homology (> 90%) with those of APPV strains that available in GenBank [19], suggesting that the newly established ELISA in this study is potential to detect antibodies against diverse strains circulating in the world. A desirable ELISA kit should be repeatable, sensitive, and specific. In this study, the intra- and inter-assay CVs of the E2-iELISA were both less than 10%, indicating the good repeatability of the test. Evaluation of the E2-iELISA showed high sensitivity, with detection limit equal to or greater than the corresponding NAb titer. In addition, the E2-iELISA displayed a high specificity, with no cross-reaction with antisera against several non-APPV viruses, including PRRSV, CSFV, BVDV, PRV, and PCV2. Furthermore, the E2-iELISA presented high agreement rate (93.7%, 178/190) with the VNT.

Previous studies have shown high APPV genome and antibody detection rates in several continents [8, 20]. In our study, 1368 serum samples collected from different pig farms in nine provinces of China were tested by the newly developed E2-iELISA, and the overall seroprevalence of APPV was 73.9% (1011/1368), suggesting wide distribution of APPV in the pig herd of China.

In conclusion, we established a suspension HEK293 cell line continuously expressing the recombinant APPV E2 protein and developed an E2-iELISA with high diagnostic sensitivity and specificity, which might be a promising tool for large-scale serological surveys.

## Data Availability

All data and materials described in this study are available.
